# Phylogenetic analysis of the bacterial intracellular R-body killer proteins indicates extensive horizontal gene transfer and signature Reb sequence motifs

**DOI:** 10.1186/s12864-026-13231-7

**Published:** 2026-07-31

**Authors:** Lennart Dörr, Robin Ghosh, Michael Schweikert

**Affiliations:** 1 Department of Biobased Materials, Institute of Biomaterials and Biomolecular Systems, University of Stuttgart, Pfaffenwaldring 57, Stuttgart, 70569 Germany; 2 Institute of Energy Efficiency in Production, University of Stuttgart, Nobelstr. 12, Stuttgart, 70569 Germany; 3 SRF Advanced Materials Innovation and Characterization (AMICA), University of Stuttgart, Pfaffenwaldring 31, Stuttgart, 70569 Germany

**Keywords:** R-bodies, Inclusion bodies, Phylogenetics, Horizontal gene transfer, Genetics

## Abstract

The fascinating spiral proteinaceous structures named ‘R-bodies’ can be produced by a number of bacterial species and are known to cause the so-called ‘killer-effect’ in paramecia. The genetic determinants of the R-bodies are the ‘*reb* genes’, which are widespread among diverse proteobacteria, presumably due to horizontal gene transfer. However, the extent of their taxonomic spread, genetic sequence diversity, and gene cluster synteny has not been analyzed exhaustively using the present genetic databases. In this study we have performed an extensive genetic survey for Reb homologous proteins, including those in previously unknown taxa. Our study reveals key amino acids of Reb protein sequences that are highly conserved and may hint at the biological role of the individual Reb proteins. We also show that the genetic synteny of *reb* gene clusters is diverse but can be clustered into distinct groups. Further, we analyze possible horizontal gene transfer events and pathways for *reb* genes and indicate context with the bacterial habitat. By identifying key aspects of R-body spread and functionality with our genetic analysis we pave the way for more targeted lab experiments that will allow R-bodies to be used for biotechnological or biomedical applications.

**Supplementary Information:**

The online version contains supplementary material available at 10.1186/s12864-026-13231-7.

## Introduction

R-bodies are peculiar spiral protein structures produced by various bacterial species. They were originally discovered through the so called ‘killer-effect’ in *Paramecium* endosymbionts *Caedibacter taeniospiralis* and *Caedimonas varicaedens* and later also in additional bacterial species, including many free-living ones [1–4]. R-bodies have unusually high chemical stability [4–7] and can be triggered to rapidly and reversibly unwind into a long rod [5, 8].

For paramecia, the so-called ‘killer-effect’ describes the ecological role of the R-body. Paramecia with R-body producing endosymbionts (‘killer paramecia’) gain a competitive advantage over paramecia without these endosymbionts (‘sensitive paramecia’). Killers occasionally release their R-body carrying endosymbionts into the environment, where they are then eaten up by sensitive paramecia. Acidification of their phagolysosome then causes the R-body to unroll, killing the sensitive *Paramecium* in this process, likely through the release of an unknown toxin [9–11].

In the legume symbiont *Azorhizobium caulinodans* R-bodies are produced when the symbiosis becomes unfavorable, leading to either the death of the host cell or elimination of the bacteria by the host [12–15]. *Pseudomonas aeruginosa* R-bodies were shown to play a role in colonization of plant and nematode hosts [16]. However, in free-living bacteria, such as *Hydrogenophaga taeniospiralis* [1, 2] and in many host-associated bacteria, like *Acidovorax avenae* [3], the functional role of the R-bodies remains largely enigmatic.

The R-body proteins found in *Cb. taeniospiralis* are the most well-researched Reb proteins and have thus served as a paradigm and reference in the field so far. They are encoded by four *reb* genes, which are present on a single 1.8 kb operon (*rebABCD*) located on an endogenous plasmid [17]. The genes *rebA* and *rebB* have been suggested to code for the structural proteins of the R-body, while *rebC* is thought to play a regulatory role. The function of *rebD* remains unknown so far [18]. In *Ps. aeruginosa* PA14 three chromosomal genes have been shown to code for the structural components of the R-body. A homologue to *rebD* with an unknown role is also present on the operon [16]. The experimental definition of alternative *reb* gene organization has also been demonstrated for the legume microsymbiont *Azorhizobium caulinodans*, where five chromosomal genes (*rebAZC1* – *rebAZC4*, *AZC_3784*) were identified to be essential for R-body formation. The expression of this *reb* cluster is controlled by a *tetR*-type regulator *rebR*, located downstream of the *rebACZ*-cluster. However, the precise molecular function of the *rebACZ* genes is unknown [12–15].

That *reb* genes are widespread among Pseudomonadota and are diverse in their genetic organizations was shown by Raymann et al. [19], who published an early genomic survey of Reb protein homologues. Their approach, however, was limited to complete genomes and focused solely on the genes of *Cb. taeniospiralis* (ctReb) as seeds for identification of Reb homologues. They also showed that two distinct synteny patterns are the most common arrangements of *reb* genes, and were defined by adjacent genes annotated as ‘hypothetical proteins’. In some cases, these translated *reb* genes share some sequence similarity with ctReb proteins [19]. However, recent studies revealed that Reb proteins can be categorized into different distinct phylogenetic groups [16, 20, 21], thereby indicating an additional layer of complexity in the gene synteny of *reb* operons.

In our recent preliminary phylogenetic analysis [21] we identified many additional homologous sequences, including in the bacterial phylum Cyanobacteria, that were not included by Raymann et al. [19] or were not available in 2013. We showed that the ‘paradigm’ ctReb proteins are phylogenetically isolated and not fully sufficient as seeds for identification of Reb protein sequences. Further, we examined the phylogenetics of R-bodies in the context of R-body morphology and extension kinetics [21].

In the current study we now extend our early phylogenetic analysis by considering every currently available sequence with Reb homology. First, we examine the genetic diversity of all Reb related sequences regarding taxonomic spread of *reb* genes and Reb protein sequence similarity. We then advance this analysis to the spread and diversity of *reb* cluster synteny patterns and complement Raymann et al. [19] in their identification of highly relevant species that are potential R-body producers and have not been put into context with R-bodies yet.

In addition, the results of this survey reveal evolutionary pathways in context with environmental occurrences of bacterial species. This is particularly interesting considering that R-body genes are very likely commonly transferred through horizontal gene transfer (HGT) [19, 21]. Finally, a look at highly conserved amino acids, as well as structuring of gene clusters gives insight into the function and role of individual R-body genes.

## Methods

### Analysis software

For our genetic survey, data analysis and visualization, we used the programming language R at versions 4.2.2 to 4.5.1 [22] using RStudio versions 2024.09 to 2025.05 [23] and the programming language Python 3.13.7 [24] with JupyterLab 4.4.4 [25]. Programming in R and Python was supported by ChatGPT versions 4o, 4o-mini, 5 and 5-mini (OpenAI) [26].

### Initial definition of Reb protein groups and selection of seeds

Based on the sequences from 56 bacterial species in our previous phylogenetic analysis of R-body genes [21], we generated a larger phylogenetic tree by using IQTREE as described in [21] that included sequences with lower homology to Reb proteins, such as those described as ‘hypothetical proteins’ of the synteny patterns by Raymann et al. [19]. This tree (not shown) served as a basis to choose representative seed sequences for defining Reb protein groups, especially for those omitted in Dörr et al. [21] for simplicity.

### Database datamining of Reb protein sequences

A list of protein sequences potentially related to known Reb proteins was assembled from the National Center for Biotechnology Information (NCBI) [27]. Sequence data and relevant annotations were collected by fetching data from NCBI manually or by using the R package *rentrez* [28]. Taxonomic information was assigned with R based on the Global Biodiversity Information Facility (GBIF) [29] by using the package *rgbif* [30, 31]. Results were curated and checked for errors or potential synonyms based on the List of Prokaryotic names with Standing in Nomenclature (LPSN) [32] database manually.

Sequences for this analysis were derived from the NCBI database and include all sequences annotated as ‘RebB family R body protein’, as well as all sequences used for our phylogenetic analysis in our recent work [21]. To identify additional Reb protein sequences that have so far remained undetected, we performed a BlastP search [33–35] with various seed sequences. The choice of seeds was based on the Reb protein groups that we assigned in our recent work [21] and selected additions based on groupings from the more detailed phylogenetic tree that was generated for an initial analysis (see above). Seed sequences were chosen, if possible, from species where R-body production has been confirmed. Seed sequences are listed in Table S1. BlastP expect threshold was set to 0.05, scoring parameters to default. ctRebC was discarded as a seed sequence since a BlastP search did not find any related sequences.

Based on the resulting dataset, additionally all sequences that are part of an identical protein group were then fetched from NCBI by using R with packages *rentrez* and *dplyr* [28, 36]. Sequences identifiable as partial sequences were excluded. Further, we removed 15 sequences of three bacterial species (*Enterobacter cloacae*, *Staphylococcus aureus* and *Acinetobacter baumannii*), as these were identical to sequences from *Pseudomonas aeruginosa* and therefore likely contaminated. Sequences assigned to non-bacterial species were also removed.

### Assignment of Reb protein groups

Each protein sequence was assigned to a Reb protein group based on multiple-pairwise alignment with each of the aforementioned seed sequences. Alignment was performed with the *Biostrings* package [37] in R. Sequences were arranged to the group with the highest identity towards its seed sequence. If sequence identity was lower than 40% towards each seed sequence, the sequence was labelled as group ‘unknown’ instead. Sequences that were assigned to the same group as the seed sequences RebB from *Cm. varicaedens* (cvRebB), RebD from *Cb. taeniospiralis* (ctRebD) and RebH from *Plesiocystis pacifica* (ppRebH) were included under ‘unknown’ for simplification, as very few sequences showed high identify towards these.

### Taxonomic spread of R-body genes

To analyze gene synteny, the list of *reb* sequences was reorganized in R to reconstruct *reb* gene cluster synteny of each assembly based on the genomic assignments of start and stop codons in the database. The respective gene group identifier was assigned to each gene of a gene synteny pattern and gaps of > 1,000 bp were marked as such in the pattern. Clusters with gaps of > 10,000 bp were split. Otherwise, gene lengths and lengths of gaps were ignored for the purpose of simplification. The data was then filtered to remove duplicates and assemblies with incomplete data. Gene synteny patterns that were identical for a single species based on their group identifiers or that had less than three *reb* related genes in each cluster were filtered. Syntenies that were not assigned at species level and were identical to a pattern of the same taxon were also excluded. Cluster structures for *Cm. varicaedens* and *Cb. taeniospiralis* were manually annotated.

Based on this list of gene syntenies, the occurrences of phylum, class and order were counted for unique species and plotted as a nested pie chart by using a custom Python script using *plotly* [38]. For each taxonomic order, the occurrences of gene groups were counted and then added to the nested pie chart as the outer ring. The sum of gene group occurrences was normalized to the count for taxonomic order.

By filtering out clusters of potentially Reb homologous genes where less than three genes are present, we reduced potential noise from sequences that might show arbitrary similarity to our Reb protein type sequences, while still retaining all potentially functional clusters.

### Co-occurrence of group-assigned *reb* genes within a *reb* cluster

The restructured and filtered table from the analysis of taxonomic spread of genes (see above) was used to analyze co-occurrence of gene groups. Clusters separated by gaps of > 10,000 were split. The number of times a gene group occurred together with another gene group per pattern was determined using a custom R script. Duplicates per pattern were excluded, while co-occurrence of identical groups were counted separately. A heatmap was generated from relative values in relation to the sum of the columns using Python with *seaborn* [39].

### Gene cluster distance matrix

To determine evolutionary relations and similarities between *reb* clusters we computed a symmetric pairwise distance matrix of protein sequence similarity between them.

Gene clusters and the corresponding protein sequences they contained were assembled and processed as described for co-occurrence above. Using MAFFT 7.526 [40, 41] running locally with L-INS-i at 1,000 iterations, we performed a multiple-sequence alignment (MSA) on all unique sequences that are part of the respective clusters. Pairwise sequence identities between all unique sequences were determined through the R package *ape* [42]. For duplicate sequences, identities were copied from their respective unique reference sequence for faster processing.

A custom distance function was then used to match similar corresponding protein sequences between gene clusters and to account for differences in gene cluster size. Proteins of both patterns were matched greedily based on lowest MSA distance. Greedy matching was chosen over the Hungarian algorithm because the resulting neighbor-joining phylogenetic tree (see below) more closely matches the distance matrix (*r* = 0.71 versus *r* = 0.62). The resulting distances were then summed up to the total MSA distance. For each unmatched gene an additional penalty equal to the lowest MSA distance of the unmatched gene to any of the matched genes was applied if the number of genes between patterns did not match.

### Visualization of gene cluster distance

To show evolutionary relations between *reb* gene clusters we generated a phylogenetic tree with a neighbor-joining approach based on the gene cluster distance matrix (see above) using the R package *ggtree* [43–47].

To test stability of the visually annotated clades (see Fig. 3) for changes in the distance matrix (see above), we compared how often sequences of each clade are consistently grouped together in the phylogenetic tree across three variations of the distance function. The distance function was modified for these variations as follows: (1) sequence matching based on the Hungarian algorithm instead of greedy matching with the R package *clue* [48]; (2) applying a fixed penalty of the maximum distance between two sequences (= 1.0) for each unmatched sequence; (3) a combination of (1) and (2).

For visualization of diversity, occurrence and similarity of *reb* gene cluster patterns, we used R to create a t-distributed stochastic neighbor embedding (t-SNE) map based on the gene cluster distance matrix (see above) using package *Rtsne* [49]. A perplexity of 40 was chosen to emphasize global structure preservation.

For the identification of distinct gene cluster groups in the resulting t-SNE map, we applied density-based spatial clustering (DBSCAN) [50] with an epsilon of 3.0 and a minimum cluster size of 15. Resulting spatial clusters were annotated with the most common genera within a cluster.

### Environmental data

Habitat and host data were assigned to bacterial species harboring R-body genes by fetching isolation sources and host data for each assembly used for the phylogenetic tree from NCBI. In addition, environmental data on species level from Microbe Atlas Project (MAP) [51, 52] and BacDive [53] were used to complement NCBI data when available. The assignments were manually curated. See Figure S1 for an overview over categories.

### Protein sequence comparison

The list of sequences used for the phylogenetic tree was separated by Reb protein groups (see above) and unique sequences of each protein group were aligned using MAFFT 7.526 [40, 41] running locally with L-INS-i at 1,000 iterations. Consensus sequences were generated for each protein group by choosing the most common amino acid at each position if the position had less than 30% gaps. For visualization purposes, four sequences of each protein group (excluding ‘unknown’) were randomly selected and aligned. For structure prediction of individual R-body proteins, we employed AlphaFold 3 via its webserver [54]. The software VMD [55] was used for visualization purposes.

## Results

### Updated taxonomic distribution of *reb* genes

Our extensive survey for Reb protein found a total of 151,976 Reb related protein sequences. However, the majority of Reb related sequences (77,260 sequences) are from *Ps. aeruginosa* due to oversampling. Out of all sequences, 9,756 were unique in either their amino acid sequence or their taxonomic classification, while 7,894 were unique in their sequence.

**Fig. 1:**
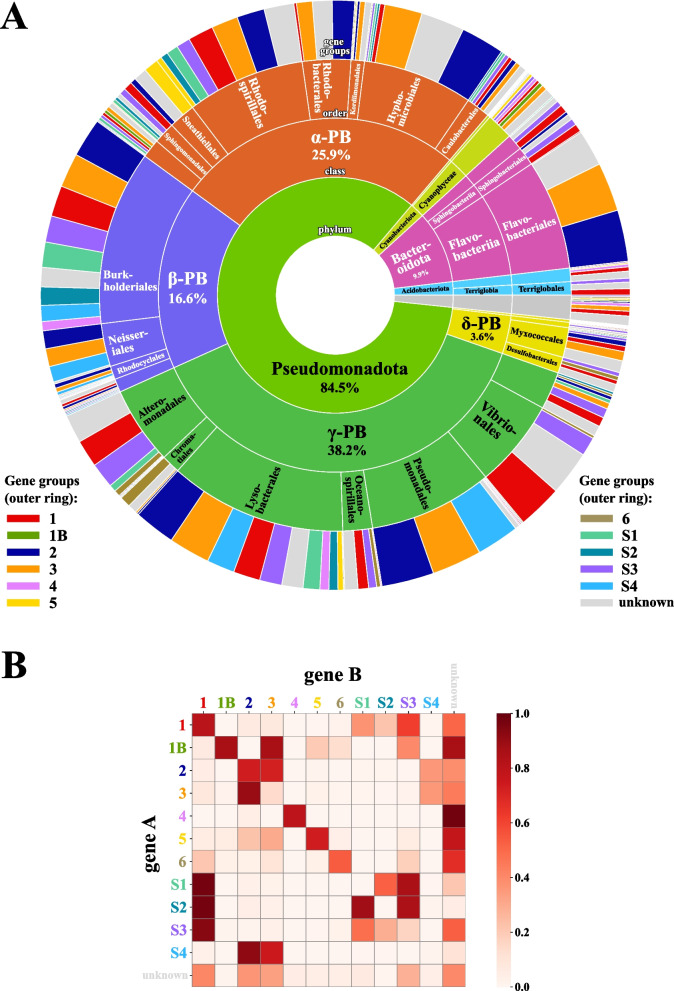
**A** Nested pie-chart showing taxonomic distribution of Reb homologous proteins by phylum (inner ring), class (second ring from the center) and order (third ring from the center). Counted were taxa that are unique and harbor ≥ 3 genes homologous to our Reb protein type sequences (*n* = 555). Taxa with *n* < 5 were grouped together with entries where the respective taxon is ‘NA’ in unlabeled categories. Outer ring, distribution of gene groups within taxonomic orders. Gene groups were counted once per unique synteny pattern in which they occurred and are relative to their related taxonomic order. Coloring of gene groups is shown in the legend and only applies to the outer ring. PB = proteobacteria. **B** Heatmap showing co-occurrence of Reb protein groups within *reb* gene clusters relative to the sum of occurrences of gene A. Multiple occurrences on a single cluster are not counted

Analysis of the distribution of *reb* genes revealed 671 unique taxa of bacteria in the NCBI database which harbor genes likely related to the production of R-bodies. Of these, there are 471 unique taxa with ≥ 3 *reb* homologous genes in at least one cluster. Taxonomic distribution by phylum, class and order is shown in Fig. 1A (the inner three rings). Apart from Pseudomonadota, Bacteroidota and Cyanobacteria, we were able to identify phyla where the presence of *reb* genes was previously unknown. Multiple species of Acidobacteriota possess ≥ 3 R-body genes, while a single unique taxon with ≥ 3 R-body genes was identified in the phyla Actinomycetota, Lentisphaerota, Planctomycetota, Streptophyta, Candidatus Tectomicrobia and Verrucomicrobiota.

In the following prokaryotic genera R-body genes are highly prevalent and present in a large number (≥ 10) of different species: *Burkholderia, Chromobacterium, Chryseobacterium, Dyella, Lysobacter, Pseudomonas, Roseibium, Thalassospira, Vibrio* and *Xanthomonas*.

### Assignment of group distribution and co-occurrence

Assigning *reb* related genes and their corresponding translated Reb proteins to groups based on protein sequence similarity enables us to perform a deeper analysis of the genes present in a *reb* cluster. Synteny analysis then supports the understanding of the role of the Reb proteins in the assembly and function of the R-body structure. While grouping of Reb proteins was determined based on protein sequences, we refer to these groups as both ‘*reb* gene groups’ and Reb protein groups depending on the respective context.

The assignment of groups is based on the 19 seed proteins that we chose based on our initial phylogenetic analysis (see above). Out of the seeds we found seven that only represent a very small number of sequences according to our criteria: cvRebB, cvRebE, ctRebB, ctRebD, mmRebA1, ppRebA and ppRebH (see Table S1 for abbreviations). In addition, ctRebC did not represent any other sequences. We therefore identified seven distinct groups (groups 1, 1B, 2, 3, 4, 5 and 6) of more closely related Reb sequences, which we refer to as ‘primary’ groups and four distinct ‘secondary’ groups (groups S1, S2, S3 and S4) more distantly related to the ‘primary’ Reb proteins. Since many other sequences could not be assigned to a specific group, we arranged them under ‘unknown’ for simplification. Seed sequences were specifically chosen to be diverse and to represent distinct groups. This is reflected in an average difference of over 22% in sequence identity across all sequences (excluding those assigned to ‘unknown’) between highest hit and second highest hit towards a specific seed.

At 25.63%, group 2 is the most common among all unique sequences per taxonomic classification. This is followed by group 1 at 17.33% and group S4 at 13.16%. Groups 1B, 5 and 6 are the rarest at < 1%.

To determine if certain *reb* gene groups commonly co-occur on a single gene cluster, we created a heatmap in Fig. 1B, which shows co-occurrence of *reb* gene groups relative to the sum of each column. The higher the co-occurrence of gene A with gene B, the higher the color intensity. Our results show that there are three gene groups: 1, 1B and 2, that commonly co-occur with other gene groups. Group 1 commonly co-occurs with S1, S2 and S3, group 1B with 3 and group 2 commonly co-occurs with groups 3 and S4. There is no common co-occurrence with other defined groups for gene groups 4, 5 and 6. The diagonal of the heatmap reveals that for groups 1, 1B, 2, 4, 5 and 6 multiple copies of a gene are commonly present on a cluster. Co-occurrences with ungrouped genes (group ‘unknown’) are especially high for groups 1B, 4, 5 and 6.

The outer ring of the nested pie-chart in Fig. 1A shows the occurrence of each group within each taxonomic order. In some taxonomic orders, such as Vibrionales and Alteromonadales (groups 1 and S3), as well as Pseudomonadales, Hyphomicrobiales, Rhodobacterales, Neisseriales and Flavobacteriales (groups 2, 3) certain co-occurring gene combinations appear to be favored greatly. In other orders, such as Burkholderiales and Lysobacterales, multiple co-occurring gene combinations are present.

### Analysis of *reb* synteny patterns

Co-occurrence of gene groups suggests the presence of distinct gene synteny patterns. To analyze these patterns, we first simplified the *reb* clusters to a sequence of group identifiers by using our previous group assignments. We identified a total of 1,406 unique synteny patterns for unique taxa with ≥ 3 *reb* genes on at least one cluster. This simplification removes duplicates with minor protein sequence differences without relevance in the context of this analysis while retaining the diversity in *reb* cluster synteny.

To determine common syntenies and evolutionary relationships between species harboring R-body genes, in particular in the context of horizontal gene transfer, we created a custom distance function that accounts for the distances between protein sequences of a gene cluster as determined through a multiple sequence alignment.

**Fig. 2:**
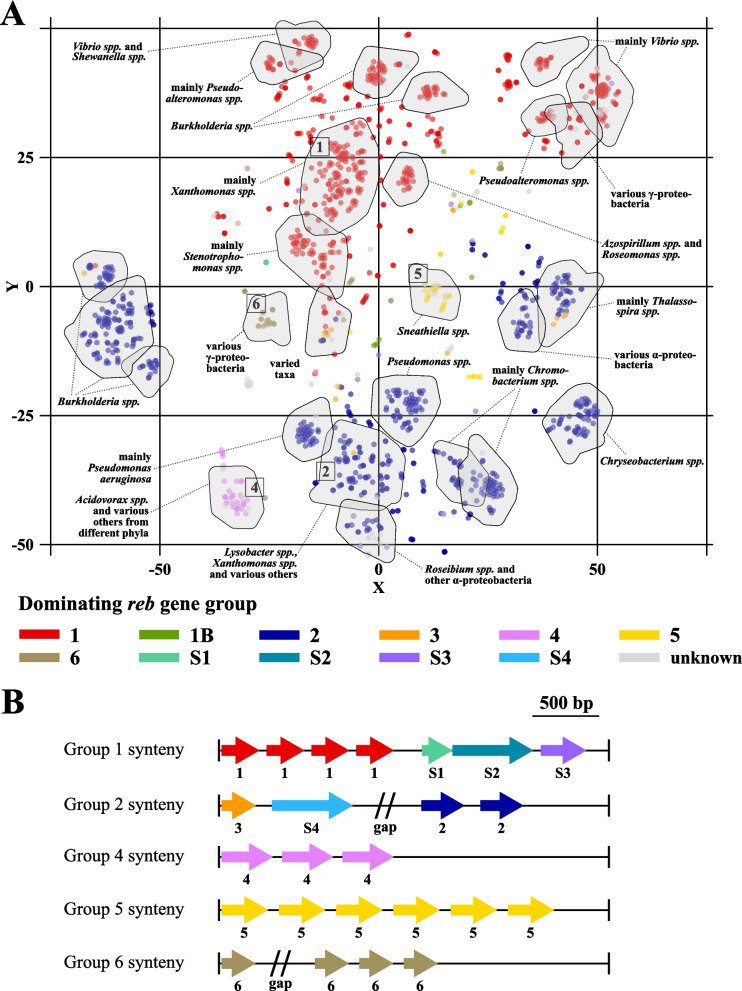
**A** t-SNE map showing spatial relationships of Reb homologous gene synteny patterns. A custom distance function was used to determine distances between *reb* gene clusters. Each dot represents a single unique *reb* gene synteny pattern. Dots are colored by the *reb* gene group that dominates the pattern with priority given to more prominent groups (e.g. when a *reb* gene cluster features two group 2 genes and two group 3 genes, the dot is colored after group 2. Group 1 is given priority over group 2.). Similar *reb* clusters are grouped together and highlighted with a grey-colored background and an outline based on DBSCAN clustering. DBSCAN clusters are highlighted with an outlined grey area according to a custom clustering script and annotated based on the respective taxa of the *reb* gene clusters they contain. Major DBSCAN clusters with the most commonly occurring synteny patterns are marked with a numbered box, corresponding to the gene map shown in (**B**). Length of genes in (**B**) are representative of the average length of genes within the respective group. Distances between genes in (**B**) are representative of the average distance between those two groups when positioned adjacent. Highlighted gaps have non-representative lengths

The resulting distances between each *reb* clusters were then used to generate both a t-SNE map and a phylogenetic tree. The t-SNE map shown in Fig. 2A displays spatial relationships among *reb* gene clusters based on protein sequence similarity. Similar *reb* clusters are grouped and highlighted with an outlined, grey-colored background based on DBSCAN clustering.

Figure 2B shows the most commonly occurring gene syntenies. Groups 1 and 2 are by far the most common, though diverse, showing subgroups where specific genes are missing, duplicated or the entire synteny is reversed (not shown), as evident by the DBSCAN clustering in Fig. 2A. The respective ‘type’ pattern displayed in Fig. 2B is marked with boxed numbers in Fig. 2A. Synteny groups 4 to 6 on the other hand form only one distinct DBSCAN cluster on the t-SNE map with some scattered isolated points for groups 5 and 6. DBSCAN clusters are commonly inhomogeneous in regard to their taxa, even though a single genus often dominates a cluster.

**Fig. 3:**
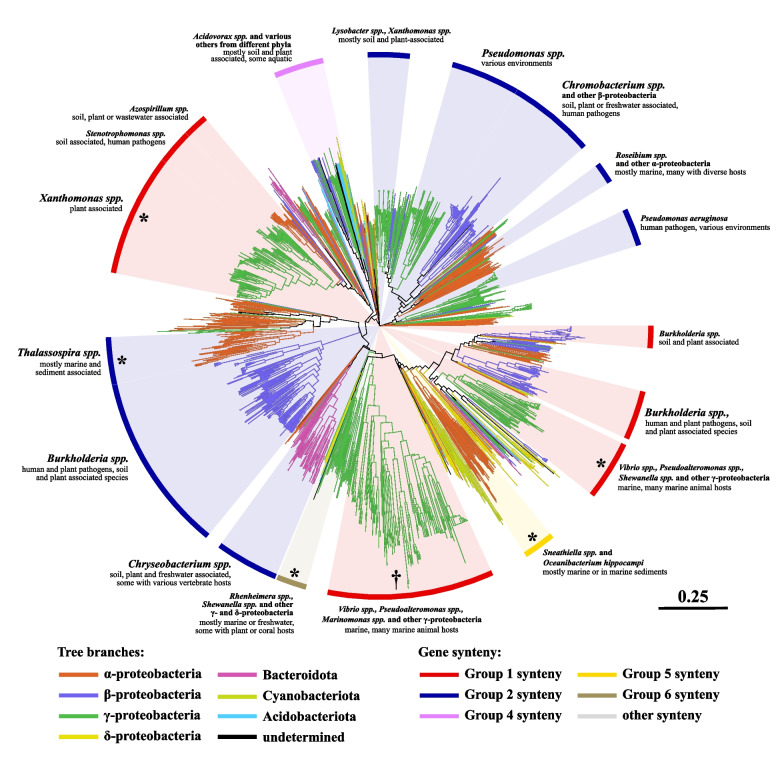
Phylogenetic neighbor-joining tree of *reb* clusters based on protein sequence similarity using a custom function. Tree branches are taxonomically colored by order (for Pseudomonadota) or class (for non-Pseudomonadota). Selected monophyletic clades are annotated by the respective taxa they contain and their respective habitats. Coloring of the clades and the outer ring is determined by the respective gene synteny group as specified in Fig. 2B. Unmarked clades are highly stable (> 90% of *reb* clusters retained across variations in the distance matrix). Clades marked with ‘*’ are stable (70 to 90% retained), ‘†’ marks only moderately stable clades (50 to 70% retained)

For the phylogenetic tree in Fig. 3 we manually highlighted selected monophyletic branches as clades based on tree distance, taxa and environment for annotation purposes. Clade color is based on the gene synteny type, while tree branches are colored taxonomically. Stability of annotated clades is indicated when *reb* clusters were retained across three variations in the distance matrix for 50 to 70% and 70 to 90% of clusters respectively (see Methods). The full phylogenetic tree, including labels for each tip that show species name, assembly ID, synteny pattern, cluster number and hosts and environments, can be seen in Figure S1.

An alternative approach to the distance function was also tested, where synteny information was incorporated by punishing positional exchange of gene, reversal of gene order and insertion of gaps of > 1,000 bp (not shown). The DBSCAN clusters (Fig. 2) and annotated clades (Fig. 3) generally showed no major differences to those based on the distance function without synteny.

### Diagnostic features of Reb protein sequences

The large collection of primary Reb protein sequences now available allow us to describe several diagnostic features.

**Fig. 4:**
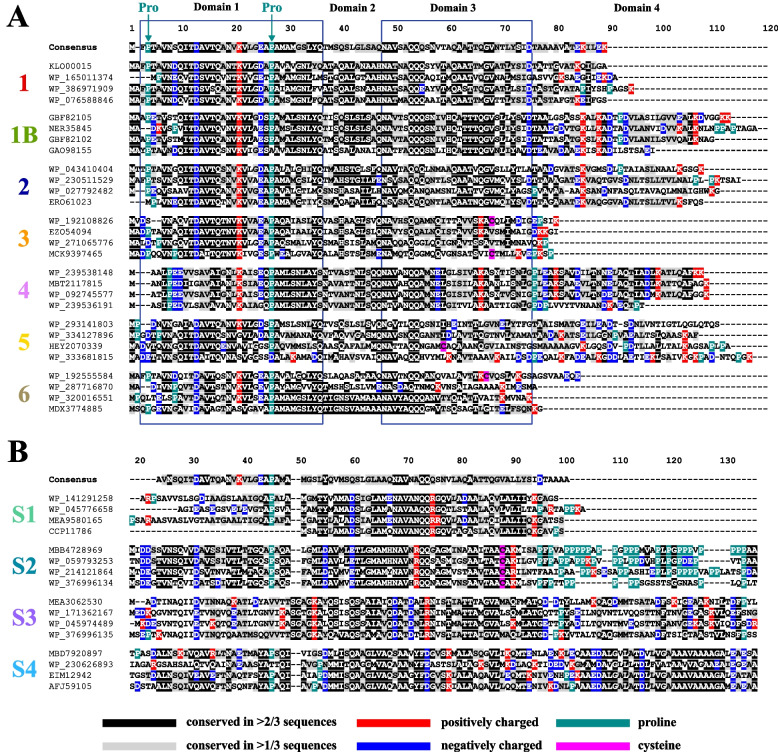
Comparison of randomly selected Reb protein sequences of different Reb protein groups. Amino acids conserved in more than 2/3 or 1/3 of consensus sequences per group are highlighted in black and grey respectively. Charged amino acids, as well as prolines and cysteines are highlighted as shown in the figure legend. **A** shows a multiple sequence alignment of the ‘primary’ protein groups. The four domains are highlighted with outlines. The consensus sequence refers to all primary Reb proteins. **B** shows part of a multiple sequence alignment of the ‘secondary’ protein groups. The consensus sequence refers to both primary and secondary Reb-proteins

First, we note that despite the well-established observation that native R-bodies cannot be fully unfolded with either strong ionic detergents (including SDS) or chaotropic reagents (e.g. 8 M urea or 6 M guanidinium chloride) [4, 6, 7] cysteine residues are rarely observed, and in the few cases where a cysteine is present, no second cysteine is present in the sequence which would allow cystine bond formation. We therefore conclude that disulfide bridges do not play a role in R-body structural stabilization. Also, aromatic residues such as phenylalanine and tryptophan are only rarely present in Reb proteins. However, although tyrosine is also rare, it is largely conserved at one position in the domain 2 we describe below (Fig. 4A).

**Fig. 5:**
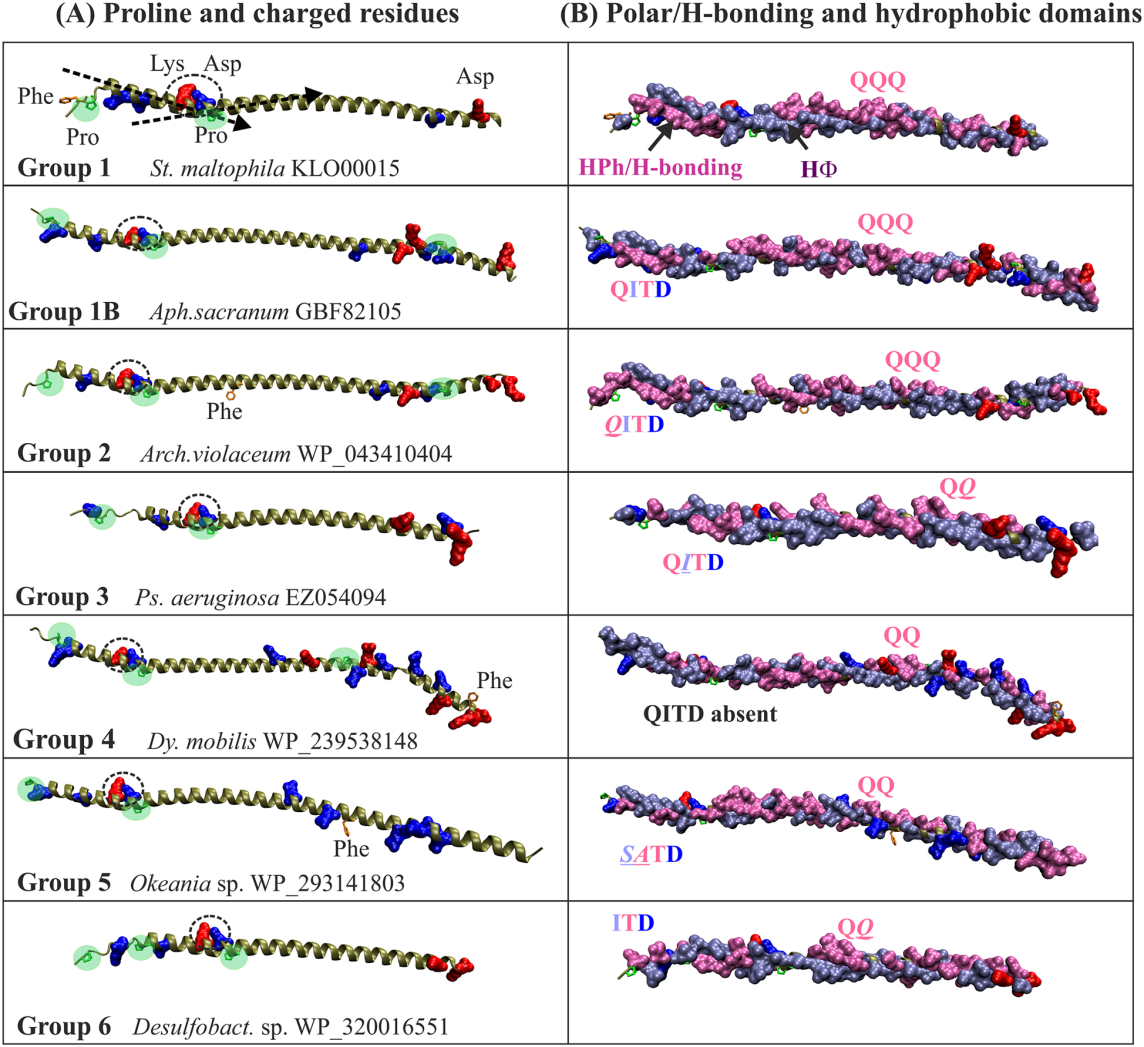
AlphaFold 3 models of randomly selected Reb proteins from the primary groups 1–6. **A** Overview of the predicted secondary structure, as well as the positions of all proline and charged residues. The positions of the phenylalanine, which is highly hydrophobic but occurs rarely in Reb proteins are also shown. **B** The same proteins shown in (**A**) shown as van der Waals surface representations, where the polar/H-bonding and hydrophobic domains are shown in mauve and purple, respectively. The positions of the diagnostic motifs QITD and QQQ are also indicated. The α-helical "kink" at the right flank of domain 1 is indicated by dashed arrows. The amino acids shown in italics and underlined indicate positions which are not highly conserved. Hydrophilic (HPh) and hydrophobic (HΦ) surfaces are also indicated

Based upon the sequence comparison and homology analysis of primary Reb proteins shown in Fig. 4A, we suggest that the sequence can be described as four structural domains. Domain 1 comprises about half (about 35 residues) of the N-terminal region and is well conserved for all Reb proteins, although the homologous regions are significantly underrepresented in Group 4 sequences. The domain is characterized by two highly conserved proline residues which flank the QITD (often DQITD) motif, which is a characteristic Reb protein signature sequence. This domain also contains a signature KXXXD/E motif which, in AlphaFold 3 models, forms an electrostatic interaction domain (see Fig. 5). The sequence comparison in Fig. 4A also shows a region which shows significant, though not extensive, homologies among Reb proteins, preceded by a sequence block which shows no common internal homologies, suggesting that it might be a "linker" region. We have therefore assigned these sequence domains to domains 3 and 4, respectively. Domain 3 often contains the characteristic NAV motif at the left flanking edge, and the QQQ (or sometimes QQ) signature motif. We note, however, that these latter sequence motifs are extensively but not absolutely conserved for all Reb proteins (see Fig. 4A, Group 2 and Group 4). In general, it is striking that domains 1–3 contain very few charged residues but are comprised mainly of either polar residues which also possess H-bonding properties, or aliphatic hydrophobic residues. By contrast, the C-terminal domain 4 is dominated by charged residues and is highly heterogenous.

We also modelled representative sequences from the primary Reb proteins using the AlphaFold 3 modelling platform. We should note that the AlphaFold platforms have never been validated for multi-subunit assemblies where the subunit stoichiometry is unknown, so the results below must be treated with caution. Nevertheless, apart from the expected prediction that the Reb proteins adopt an α-helical structure, which was previously reported for smaller sampling groups [19, 56], we also observed two new structural features. First, as mentioned above, the highly conserved charged residues in the KXXXD/E motif of domain 1 are close enough to experience a strong electrostatic interaction. (Fig. 5, left hand panels), facilitated by the "kink" in the α-helical secondary structure due to the neighboring, highly conserved proline residue (see [57, 58]). Secondly, due to the 3.6 residue/turn property of an α-helix, the amino acids in domains 1–3 appear to cluster in small domains with either polar, H-bonding or hydrophobic character, respectively (Fig. 5, right-hand panels). Interestingly these small polarity domains seem to be rotationally and alternately distributed along the α-helix.

The ‘secondary’ Reb-protein groups have some similarities in their sequences to those of ‘primary’ Reb protein groups but differ in key aspects (see Fig. 4B). In Group S1 only the diagnostic motifs of domain 3 are relatively well conserved. Group S2 shows the most similarity to the ‘primary’ protein groups in domains 1 and 3 but possesses an extended proline-rich C-terminal region. Group S3 lacks the characteristic proline and the motifs of domain 2 and 3. S4 is the most different and has an extended N-terminal region, which is rich in both proline and charged amino acids. It also lacks many of the diagnostic motifs of the domains and has an extended C-terminal region which bears no resemblance to domain 4 of the primary sequences.

## Discussion

Our study reveals that R-body proteins are extensively distributed across a large variety of bacterial phyla (Fig. 1A) and are present in many species exhibiting bacterial-eukaryotic interactions, as well as in many free-living bacteria. Notably Reb protein genes are found in well-known plant pathogens (*Burkholderia* spp., *Acidovorax* spp., *Xanthomonas* spp., *Agrobacterium vitis*, *Ralstonia syzygii* and *Trinickia caryophylli*)*,* plant-beneficial bacteria (*Azospirillum* spp., *Bradyrhizobium* spp., *Paraburkholderia* spp., *Lysobacter* spp. and *Pseudomonas* spp.), pathogens of marine eukaryotes (*Kordia algicida* and *Vibrio* spp.), symbionts of marine eukaryotes (*Pseudovibrio* spp.), and also human pathogens (*Vibrio* spp.*, Chryseobacterium indologenes*, *Inquilinus limosus*, *Chromobacterium violaceum*, *Stenotrophomonas maltophilia*, *Burkholderia* spp. and *Pseudomonas* spp.). Thus, R-bodies have both pivotal, as well as highly variable roles in Gram-negative bacteria, and we suspect that their multifaceted roles in nature may still be largely unexplored. However, we note also that even though in many cases *reb* gene synteny and protein sequence are well conserved, the physical presence of Reb proteins has still to be confirmed experimentally for many species. This is not trivial, since R-bodies only reside in a fraction of the population and may often require environmental stress to trigger R-body production [16, 21].

Our detailed analysis of Reb proteins by sequence and synteny strongly suggests that the ‘primary’ Reb protein groups 1, 1B, 2, 4, 5 and 6 (see Fig. 2B) encode the R-body structural proteins, since they generally share high identities to the structural proteins proposed previously for *Cb. taeniospiralis* (ctRebA and ctRebB) [18], as well for *Ps. aeruginosa* (paRebC (RebP1) and paRebD (RebP2)) [16]*.*

Group 3 proteins have previously been shown to not be required for R-body synthesis or structure so far [16, 18]. However, group 3 genes are commonly found together with those of the group 2 and well conserved within group 2 synteny patterns, suggesting that they are relevant for R-body assembly.

Sequence homology is only very weak for the ‘secondary’ Reb proteins S1 to S4, which are highly diverse and not as consistently preserved or even fully absent on distinct *reb* cluster syntenies. Some of these proteins share sequence similarities to ctRebC from *Cb. taeniospiralis*, which may also have a regulatory role in R-body assembly [18]. In fact, one member of group S4, paRebB (rapA in [16]) of *Ps. aeruginosa*, was reported to be present in isolated R-bodies in similar amounts to the group 2 proteins and appears to be associated with one of them, implying a non-stoichiometric role in the assembly process.

The seed sequences for our group assignment were chosen based on distinct clustering within a phylogenetic tree in a preliminary analysis presented elsewhere [21] and were deemed suitable to cover the vast diversity of Reb proteins. We note, however, that assigning Reb protein groups in this way can introduce bias, particularly for individual Reb proteins with low sequence homologies towards a specific seed. Nevertheless, our results in the synteny analysis and sequence comparison show that the Reb protein groups we defined here are biologically meaningful, as they match well with synteny groupings and characteristic sequence motifs (see Fig. 2).

The approach in our study has been simplified by ignoring the ‘hypothetical proteins’ which show no homology to Reb proteins (HP1.1, HP2.2, HP2.3 and HP2.4), but which still colocalize with *reb* genes [19]. Significantly, many of these were putatively annotated as RNA polymerase sigma-factors or other transcriptional regulators, suggesting a regulatory role. In some cases, evidence supports the fact however that non-homologous genes can play a role in the regulation of R-body formation [12, 16]. Identifying such genes by phylogenetic analysis is difficult, as the co-localization of a gene without additional information (e.g. homology or a distinct synteny pattern) may be meaningless, and would contribute significantly to noise in the dataset.

In addition to the characteristic synteny types that we defined, our study also reveals the existence of many unique syntenies (not shown) that only occur in a very limited number of *reb* gene clusters. These *reb* clusters are either from under-sampled bacterial groups or must be highly specialized, like the *reb* clusters of *Cb. taeniospiralis* and *Cm. varicaedens*.

In our study, we reduced the complexity of a phylogenetic tree of individual Reb proteins to a tree that displays evolutionary relationships between distinct *reb* clusters per taxon. This approach is better suited to revealing evolutionary pathways, in particularly by horizontal gene transfer (HGT), since *reb* clusters often appear to be commonly distributed between species as a complete unit, *reb* genes that are part of a single *reb* cluster can be phylogenetically highly scattered individually and *reb* gene synteny is commonly shuffled around. We note, however, that building a reliable, stable and fully accurate phylogenetic tree of Reb related protein sequences or *reb* gene clusters based on current data is not satisfying at present, since some sequences and gene syntenies can show considerable variability, which may obscure the relations, in particular between distantly related clusters. This is reflected in the fact that some clades do show instability towards changes in the distance matrix (Fig. 3). Further, the neighbor-joining algorithm is unable to perfectly transfer the distance matrix to the topology of a phylogenetic tree (here *r* = 0.71). While a phylogenetic tree is more readable and useful for annotation purposes, the complementary t-SNE map that we provided here is better suited to display relations between gene clusters. In particular, while Fig. 2A groups gene clusters of the same synteny group together consistently, this is not always the case in Fig. 3, where early evolutionary relations between the major clades are unreliable. Nevertheless, while a detailed interpretation of the visualizations of our distance matrix should be treated with caution, they are useful in identifying distinct groups of *reb* gene clusters and in tracing out general evolutionary pathways.

Our data clearly shows that the evolutionary history of *reb* gene inheritance has been vastly shaped by horizontal gene transfer (HGT). For instance, among the genera where many species feature R-body genes, such as *Burkholderia* spp., *Xanthomonas* spp. and *Pseudomonas* spp., there are many taxa well known for commonly acquiring new genes via HGT [59–63].

A particular striking example of HGT can be observed for the *Burkholderia* spp.* reb* clusters, which are spread across the entire tree, with a large clade encompassing *reb* clusters featuring group 2 synteny clusters, while another clade features group 1 synteny clusters. Some individual clusters are even located within other clades. Many *Burkholderia* spp. have two clusters of *reb* genes in their genome, where some feature one with group 1 and one with group 2 synteny. Correspondingly, phylogenetic trees based on 16S rRNA for *Burkholderia* (e.g. [64].) do not align well with the phylogenetic trees derived from *reb* cluster proteins. For some species of *Burkholderia*, even clusters from different strains of the same species are observed to be scattered. Taken together, these data imply that within the genus *Burkholderia*, there is a high HGT rate for *reb* clusters, while simultaneously *reb* clusters of some strains must have been acquired horizontally from other genera.

We have also observed that many *Ps. aeruginosa reb* clusters are located in a single clade that is more distantly related to those of other *Pseudomonas* sp., again implicating HGT events. We also observed that the clades encompassing many marine bacteria (e.g. *Vibro* spp., *Pseudoaltermonas* spp. and *Marinomonas* spp.), which exclusively feature γ-proteobacteria, are of interest, since they often possess two different group 1 synteny *reb* clusters located separately on their genome. Bacteria in marine environments have been shown to have high rates of HGT [65], which are facilitated by bacterial host environments, e.g. sponges, corals, squids or crustaceans. It has been shown that the single *reb* cluster of *V. nigripulchritudo* is sufficient for complete R-body production [66], though a detailed comparison with other wild-type R-bodies is still lacking. Assuming full functionality of these R-bodies, we may deduce that two *reb* clusters in these groups may not be essential for R-body formation, although we cannot eliminate the possibility that under some conditions, R-body hybrids formed from proteins differentially expressed from both clusters may be present. In general, however, we observe that multiple *reb* clusters within the same genome are uncommon.

Finally, for cyanobacteria, which are amongst the most abundant bacterial species on the planet, only a few *reb* clusters are present in the database (e.g. in *Oscillatoria*, *Crocosphaera*, *Symploca* and *Aphanothece*). Most of these clusters group together in a single, tree region, though with limited stability.

In summary, we observe that HGT of *reb* genes is extensive and that genes are sometimes scattered to very distant phyla, though most *reb* gene HGT events occur between taxonomically closely related taxa. Also, whereas some clades are exclusive to or associated with a specific environment, syntenies do not appear to be directly related to the bacterial habitat.

Our sequence analysis has now established new diagnostic motifs (QITDN, KXXXD/E, NAV, QQ(Q)) for primary Reb proteins, which may facilitate their rapid identification, and which probably have an important structural role in R-body assembly. The rarity of cysteine residues in all the Reb sequences essentially eliminates the possibility that disulfide bridges are responsible for a previously suggested [6, 18] covalent linkage between R-body protein subunits.

The AlphaFold 3 models of randomly chosen representative sequences yield new insights for the interpretation of the sequence data. In particular, the proline-mediated "kink" in domain 1 facilitates the electrostatic juxtaposition between charged residues of the conserved KXXXD/E motif, suggesting an important role in the R-body assembly. However, the most intriguing result of the AlphaFold 3 modelling, is that the amino acids of domains 1–3 are arranged in polar or hydrophobic domains, respectively. This is very similar to what is observed for subunit interfaces in proteins in general and was first observed for the α_1_β_1_ and α_1_β_2_ domains of hemoglobin [67]. We therefore suggest that domains 1–3 have a key role in the subunit assembly of the R-body. The highly polar, charged C-terminal domains imply an important role in the well-known acid-induced unrolling of R-bodies, as was first suggested by Polka and Silver [68].

The persistent structural features (flanking prolines and charged amino acid electrostatic interaction domain 1, and the rotationally distributed hydrophilic and hydrophobic domains along the length of the α-helix) of Reb proteins from highly diverse species suggests that the fundamental principle which allows rapid unrolling of the R-bodies (‘the killer effect’) has been unaffected by HGT. Thus, the elucidation of even a single R-body structure at the atomic level will probably facilitate reliable modelling of this very large family of biomolecular machines.

## Supplementary Information

Supplementary Material 1.

Supplementary Material 2.

## Data Availability

The raw sequence datasets and a high-resolution version of Figure S1 have been deposited at DaRUS, the data repository of the University of Stuttgart and are publicly accessible at 10.18419/DARUS-5753.
