# Supplementary material for: Phylogenetic analysis of the bacterial intracellular R-body killer proteins indicates extensive horizontal gene transfer and signature Reb sequence motifs
Source: BMC Genomics. 2026 Jul 31;27:646. doi: 10.1186/s12864-026-13231-7 (PMC13430693; doi:10.1186/s12864-026-13231-7)
Supplement: Supplementary file 1 — Supplementary Material 1. [file 12864_2026_13231_MOESM1_ESM.docx]

**Table S1.** List of Reb protein group type sequences used as seeds for the initial BlastP search. Amino acid length and the respective protein group assignments are given.

| **Name** | **species** | **Protein ID** | **Length** | **Group** |
| --- | --- | --- | --- | --- |
| htRebA | *Hydrogenophaga taeniospiralis* | WP_068173291.1 | 98 | 1 |
| cvRebC | *Caedimonas varicaedens* | - | 133 | 1B |
| paRebC | *Pseudomonas aeruginosa* | WP_003108980.1 | 106 | 2 |
| paRebA | *Pseudomonas aeruginosa* | WP_003090893.1 | 80 | 3 |
| aaRebB | *Acidovorax avenae* | WP_013596746.1 | 103 | 4 |
| rcRebE | *Rhodocista centenaria* | WP_012567175.1 | 107 | 5 |
| etRebA | *Exilibacterium tricleocarpae* | WP_142905012.1 | 80 | 6 |
| htRebE | *Hydrogenophaga taeniospiralis* | WP_068173298.1 | 65 | S1 |
| htRebF | *Hydrogenophaga taeniospiralis* | WP_068173300.1 | 184 | S2 |
| htRebG | *Hydrogenophaga taeniospiralis* | WP_068173302.1 | 113 | S3 |
| paRebB | *Pseudomonas aeruginosa* | WP_003108962.1 | 210 | S4 |
| ctRebB | *Caedibacter taeniospiralis* | WP_011178427.1 | 105 | unknown |
| ctRebD | *Caedibacter taeniospiralis* | WP_011178426.1 | 81 | unknown |
| ctRebC | *Caedibacter taeniospiralis* | WP_011178482.1 | 114 | unknown |
| cvRebB | *Caedimonas varicaedens* | - | 119 | unknown |
| cvRebE | *Caedimonas varicaedens* | - | 129 | unknown |
| mmRebA1 | *Marinomonas mediterranea* | WP_013661812.1 | 66 | unknown |
| ppRebA | *Plesiocystis pacifica* | WP_006976348.1 | 114 | unknown |
| ppRebH | *Plesiocystis pacifica* | WP_006976341.1 | 111 | unknown |
