# Supplementary material for: Phylogenetic analysis of the bacterial intracellular R-body killer proteins indicates extensive horizontal gene transfer and signature Reb sequence motifs
Source: BMC Genomics. 2026 Jul 31;27:646. doi: 10.1186/s12864-026-13231-7 (PMC13430693; doi:10.1186/s12864-026-13231-7)
Supplement: Supplementary file 2 — Supplementary Material 2. [file 12864_2026_13231_MOESM2_ESM.docx]

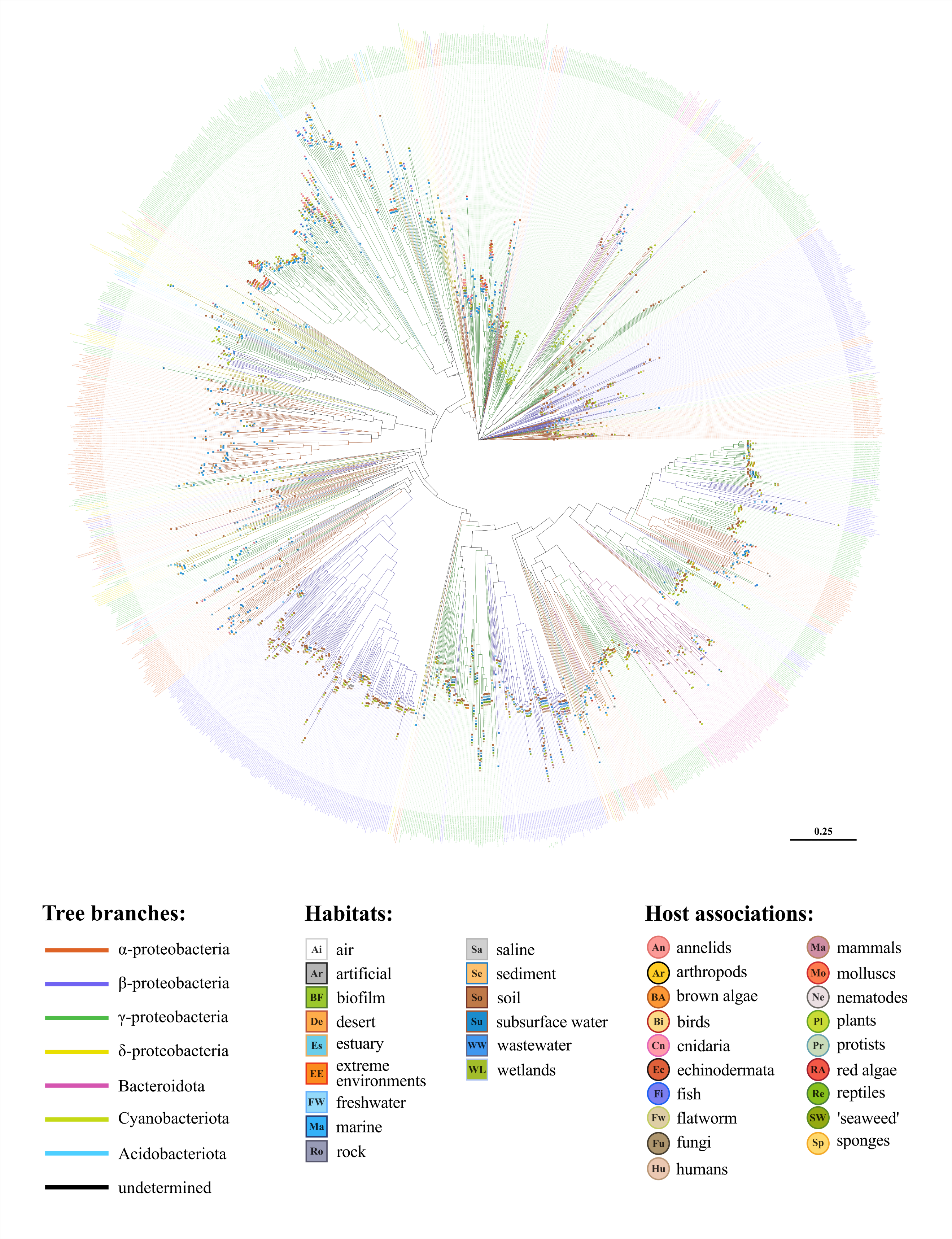


**Fig. S1.** Phylogenetic neighbor-joining tree of reb clusters based on protein sequence similarity and a custom function used to describe changes in gene synteny. Tree branches are taxonomically colored by order (for Pseudomonadota) or class (for non-Pseudomonadota). More detailed version with labels of which Fig. 3 is based on. Labels display species name (if available), assembly ID and gene synteny, as well as environments of the respective bacterial species displayed by icons. A high-resolution version of this graphic is available via DaRUS, the data repository of the University of Stuttgart.
